# Genome Sequences of a Novel HIV-1 Circulating Recombinant Form, CRF55_01B, Identified in China

**DOI:** 10.1128/genomeA.00050-12

**Published:** 2013-01-24

**Authors:** Xiaoxu Han, Minghui An, Weiqing Zhang, Weiping Cai, Xi Chen, Yutaka Takebe, Hong Shang

**Affiliations:** a Key Laboratory of AIDS Immunology of Ministry of Health, Department of Laboratory Medicine, The First Hospital of China Medical University, Shenyang, China; b Infectious Diseases Department, Guangzhou No. 8 People's Hospital, Guangzhou, China; c Hunan Provincial Center for Disease Control and Prevention, Changsha, China; d AIDS Research Center, National Institute of Infectious Diseases, Tokyo, Japan

## Abstract

We report here a novel HIV-1 circulating recombinant form (CRF55_01B) composed of CRF01_AE and subtype B, with four recombination breakpoints in the *pol* gene. CRF55_01B was identified from three epidemiologically unlinked men having sex with men (MSM) in China, suggesting the ongoing generation of recombinants involving CRF01_AE and subtype B lineages among MSM in China.

## GENOME ANNOUNCEMENT

A high level of genetic diversity is a hallmark of human immunodeficiency virus type 1 (HIV-1). HIV-1 group M, a major (main) group of HIV-1 strains responsible for the HIV pandemic, consists of 11 subtypes and sub-subtypes (A1, A2, B, C, D, F1, F2, G, H, J, and K), and 54 circulating recombinant forms (CRFs) have been reported to date (http://www.hiv.lanl.gov). Wide cocirculation of and dual infection with CRF01_AE and subtype B in various geographical regions in Asia led to the emergence of various novel CRFs, including CRF15_01B (1) and CRF34_01B (2) in Thailand, CRF33_01B (3), CRF48_01B (4), CRF53_01B (5), and CRF54_01B (6) in Malaysia, CRF51_01B (7) in Singapore, and CRF52_01B (8) in Thailand and Malaysia. Here, we describe the genome sequences of a novel CRF (designated CRF55_01B) isolated from three epidemiologically unlinked men having sex with men (MSM) in China.

Plasma was collected from three recently infected MSM in two different regions in China. Near-full-length genome (NFLG) sequences (9.0 kb) were determined from plasma RNA, using a single-genome amplification method with two sets of primers designed for determination of 5′ and 3′ halves of the HIV-1 genome sequence (9, 10). Amplicons were directly sequenced using the internal walking primers with an ABI 3730XL Sanger-based genetic analyzer. Sequences were assembled using the Sequencher program and manually edited using the BioEdit program. The study was approved by the ethics committees of China Medical University and the hospitals that participated in this study.

The three NFLGs of CRF55_01B were 8,925, 8,952, and 8,950 bp for strains 10CN.HNCS102056, 11CN.GDDG095, and 11CN.GDDG318, respectively, spanning the noncoding region (NCR), the *gag, pol, env, tat, rev, vif, vpr, vpu*, and *nef* genes, and a 5′ part of the 3′ long terminal repeat (LTR). These three strains formed a distinct monophyletic cluster distantly related to all known HIV-1 subtypes/CRFs. Bootscanning and informative site analyses (11) identified four unique recombination breakpoints between CRF01_AE and subtype B at the nucleotide positions 3060, 3329, 3767, and 4453 (relative to the HXB2 genome) that were shared among all three strains. Subregion tree analyses further confirmed the parental origin of each region of the recombinant genome as follows: region I (HXB2 nucleotides [nt] 790 to 3059), CRF01_AE; region II (HXB2 nt 3060 to 3328), subtype B; region III (HXB2 nt 3329 to 3766), CRF01_AE; region IV (HXB2 nt 3767 to 4451), subtype B; and region V (HXB2 nt 4453 to 9613), CRF01_AE. The recombinant structure is distinct from any known CRFs reported to date. Subregion tree analyses also revealed that the subtype B regions were of U.S.-European origin, not in the subtype B′ (Thailand variant of subtype B) (12, 13) lineage associated with bloodborne epidemics in Asia (14), whereas the CRF01_AE regions were associated with Thailand CRF01_AE radiation and were not related to the CRF01_AE variants (clusters 1 and 2) recently identified among MSM in China (9).

CRF55_01B is one of the first CRFs circulating principally among a population of MSM. The only other example known to date is CRF51_01B, recently isolated from MSM in Singapore (7). The emergence of CRF55_01B suggests the ongoing generation of novel recombinant strains and an active transmission network(s) among MSM in China, where HIV-1 epidemics among MSM are surging rapidly (15).

### Nucleotide sequence accession numbers.

The sequences are available in Genbank under the accession numbers JX574661 to JX574663.
